# A Comprehensive Design-to-skin Pipeline to Fabricate Polymeric Microneedles Using Ultrahigh-resolution 3D Printing

**DOI:** 10.1007/s11095-025-03936-x

**Published:** 2025-11-11

**Authors:** Francesco La Malfa, Isabella A. van Hulst, Ferry Ossendorp, Urs Staufer, Koen van der Maaden

**Affiliations:** 1 Department of Precision and Microsystem Engineering, Delft University of Technology, Delft, 2628 CD The Netherlands; 2 Department of Immunology, Leiden University Medical Center, Leiden, 2300 RC the Netherlands

**Keywords:** 2-photon polymerisation, (intra)dermal drug delivery, Microneedle array, Moulds, PDMS, Skin penetration

## Abstract

**Objective:**

Microneedle technologies have emerged as a promising approach to improve intradermal drug delivery. This study presents a comprehensive workflow for fabricating polymeric microneedle arrays utilising ultrahigh-resolution 3-dimensional (3D) printing and silicone mould fabrication.

**Methods:**

In this work, an extensive toolbox with over 75 distinct microneedle designs was created and sequentially fabricated from acryl using our workflow based on ultrahigh-resolution 3D printing.

**Results:**

The microneedle design parameters included obelisk and cone-like shapes, various lengths, base and tip diameters, and different densities. We systematically assessed the optimal design parameters for effective penetration of *ex vivo* human skin explants.

**Conclusion:**

Our workflow, combined with application in an *ex vivo* human skin model, allows systematic comparison of multiple microneedle design parameters for efficacy. This work demonstrates the potential of this systematic modelling and ultrahigh-resolution 3D printing approach to optimize microneedles for intradermal biomedical applications, including therapeutic cancer vaccination.

**Graphical Abstract:**

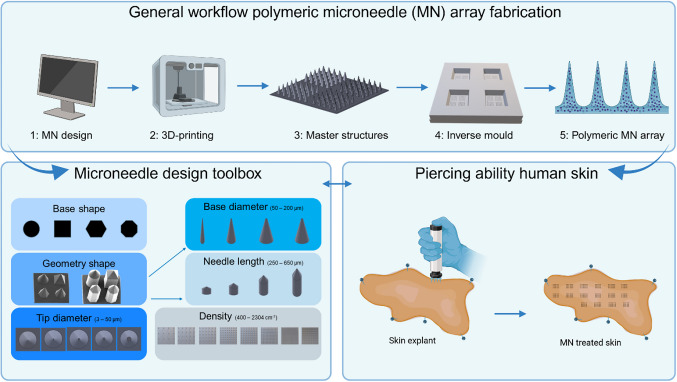

**Supplementary Information:**

The online version contains supplementary material available at 10.1007/s11095-025-03936-x.

## Introduction

The skin is a critical barrier against external environmental threats, protecting underlying tissues from pathogens, chemicals, and physical injuries. It comprises multiple layers: the stratum corneum (SC), an almost impermeable layer, the epidermis and the dermis, and the subcutaneous fat layer. The skin’s outermost layer, the SC (15—20 µm), is particularly important for its barrier properties. [1, 2] Beneath the SC lies the viable epidermis (130—180 µm) and dermis (1000—1200 µm), which contain several skin-resident immune cells, especially antigen-presenting cells such as dendritic cells (DCs) [1, 3]. Thereby, the skin is a first-line immune sensing organ. Not surprisingly, the skin can be utilised very effectively to induce protective immunity. Compared to classical (*i.e.*, intramuscular and subcutaneous) administration routes, vaccinations via the skin can result in a 3- to 20-fold dose-sparing [4] as a result of increased immunogenicity [5–7]. This is especially beneficial in the case of emerging infectious diseases and pandemic situations when vaccine antigens are scarce. Moreover, the skin is especially suitable for T cell-mediated (cancer) immunotherapy due to the high number of DCs, as they are key players in the induction of T cells [8–10].

In pursuing advanced intradermal drug delivery, microneedle technologies have emerged as promising delivery systems. A microneedle is a submillimetre-scale needle-like structure ranging from tens to hundreds of micrometres in width with a length below one mm. They are engineered to facilitate controlled and targeted delivery of therapeutic agents or vaccines through the SC into the epidermis [5, 11–15]. Microneedle technologies can be applied for many therapeutic interventions, including anti-infectious, -cancer, -allergy, and -autoimmune therapies [16, 17]. One of the main advantages of microneedles is their potential for self-administration in a minimally invasive and pain-free manner because they do not reach the pain receptors in the dermis [18].

The clinical potential of microneedles, particularly in vaccination and immunotherapy, relies on their precise design, fabrication approach, insertion method, and mechanism of drug release. Critical factors such as microneedle density, geometry, length, tip diameter, and overall array layout significantly influence the microneedles’ ability to reliably penetrate the skin barrier and deliver therapeutic agents to targeted layers. In parallel, fabrication methods must ensure structural integrity and reproducibility, while insertion technique and drug release mechanisms further dictate delivery efficiency. Several studies have looked into these factors via computational modelling; however, the impact of microneedle design parameters on human skin penetration has not been extensively studied or documented. Our study tackles the lack of systematic studies on the effect of microneedle design on the skin piercing ability. Conventional fabrication techniques (*i.e.*, chemical wet etching [19–21] and UV lithography [22–24]) for microneedle manufacturing are time-consuming and make it difficult to change/adjust one design parameter at a time. Therefore, most design studies vary multiple microneedle parameters simultaneously, making it challenging to determine the effect of a single parameter on skin piercing.

To overcome these challenges, ultrahigh-resolution 3D printing by 2-photon polymerisation (2PP) might be a viable choice. 2PP 3D printing offers several advantages over other techniques: 2PP allows exact control over the fabrication process at the micro- and nanometre scale level while lowering the expenses associated with different techniques [25]. This capability gives rise to the possibility of designing microneedles tailored to specific applications with precise characteristics. Unlike traditional 3D printing methods, which rely on filament-based or single-photon absorption processes, 2PP uses ultrashort laser pulses to polymerise submicron structures from photosensitive resin. This process involves the simultaneous absorption of two photons, in which the energy is absorbed by the photo-initiator molecules present in the resin. The selective polymerisation is given by the dimension of the volume (voxel) where the intensity of the laser beam is sufficiently high to allow the two-photon absorption process to happen, which can be three-dimensionally processed. This methodology enables precise control over microneedle design parameters while maintaining nanometre-scale resolution, facilitating the production of sharp and tailored microneedles. By varying a single microneedle design parameter at a time, it becomes possible to systematically investigate its individual effect on skin penetration.

Although ultrahigh-resolution 3D printing opens many possibilities for future microneedle-mediated drug delivery, 2PP printed arrays are currently not suitable for use in living organisms. 2PP uses photosensitive resins that, once cured, may not have the necessary mechanical strength and chemical properties required for skin applications. Furthermore, the materials used in 2PP are mainly optimised for their photo-polymerisation properties rather than their mechanical or biocompatible properties. For microneedles, it is essential that the material is biocompatible, non-toxic, non-immunogenic (prevents unwanted allergic skin reactions), and maintains its integrity in the biological environment. Therefore, the first aim of this study was to set up a general workflow to produce polymeric microneedles utilising 2PP in combination with a moulding process that exploits polydimethylsiloxane (PDMS) to produce an intermediate, inverse mould. This approach decouples, to a large extent, the material selection for the microneedles from the process that defines their form [26].

The second aim was to generate a toolbox of different microneedle designs suitable for producing solid microneedle arrays. Several parameters regarding microneedle design were included in this toolbox, including needle geometry (cone- and obelisk-like structures), shape (circular, tetragonal, hexagonal, and octagonal), length, base diameter (and hence the cutting edge), and tip diameter.

The third and final aim was to determine the critical microneedle design parameters (from the toolbox) for human skin penetration by testing all fabricated microneedle arrays. To our knowledge, this is the first study systematically investigating the effect of microneedle design on *ex vivo* human skin piercing. Herewith this study shows the applicability of 2PP for microneedle fabrication processes and presents a general workflow for the generation of polymeric microneedles.

## Materials and Methods

### Materials

Silicon (Si) substrates and IP-Q, a proprietary photosensitive acrylate resin used to fabricate all the two-photon-polymerised structures, were purchased from Nanoscribe GmbH & Co. KG, Karlsruhe, Germany. Acetone, isopropyl alcohol (IPA), propylene glycol monomethyl ether acetate (PGMEA), 3-(trimethoxysilyl)propyl methacrylate (MAPTMS), and Trichloro (1H,1H,2H, 2H-perfluorooctyl) silane (TPFS), polyvinyl alcohol (PVA) (Mw 9–10 kDa, 80% hydrolysed), and sodium carboxy-fluorescein were purchased from Sigma-Aldrich, Germany. Polydimethylsiloxane (PDMS) (Silicone Elastomer Flowable Sylgard 184) was purchased from Farnell, Netherlands. Phosphate-buffered saline (PBS) (137 mM NaCl, 2.7 mM KCl, 10 mM Na_2_HPO_4_, 1.8 mM KH_2_PO_4_ and pH 7,4) was obtained from Fresenius Kabi, Huis Ter Heide, Netherlands, and epoxy glue was from Bison International B.V., Goes, the Netherlands. Trypan blue (Mw 960.81 g/mol, 0.4% (w/v), sterile-filtered) was purchased from Millipore Sigma, Netherlands.

### Computer-Aided Design (CAD) and 2-photon Polymerisation Step

The fabrication of the microneedle array starts with the design of the different master templates that were printed in the following step. SolidWorks (Dassault Systèmes SolidWorks Corp., Vélizy-Villacoublay, France) was used as CAD software for designing the files that were imported into the proprietary Describe software (version 2.7, Nanoscribe GmbH & Co. KG, Karlsruhe, Germany) as ‘.stl’ (standard triangle language) file extensions. Importing such files into the software allows for the optimisation of printing parameters and the creation of a ‘.gwl’ (general writing language) file that contains all the printing information and the total (simulated) time for the laser writing. While parameters, such as slicing (dividing the templates into vertically stacked planes in the z direction) and hatching (dividing the slide planes into voxel trajectory lines in the xy plane), followed the standard recipe from Nanoscribe GmbH, parameters such as Block dimensions and Offset have been optimised to reduce the duration of the printing process. The system employs a femtosecond fibre laser source with a wavelength of 780 nm, repetition rate of 80 MHz, pulse length of 100 ps; the maximum laser power is 50 mW (corresponding to 100% laser power). A 10 × microscope objective, with a numerical aperture (NA) of 0.30, focused the laser on the samples. The configuration used was the 'dip-in laser lithography' (DiLL), in which the lens is directly dipped into the photoresist. Finally, the IP-Q resin has been used as a photoresist: IP-Q is a negative tone-methacrylate-based photoresist. The designs were imported to the Nanoscribe Photonic Professional GT2 (Nanoscribe GmbH & Co. KG, Karlsruhe, Germany) software (Nanowrite, version 1.10.5) for the 2-photon polymerisation process. At the beginning of this procedure, laser power and scan speed were optimised for printing, namely 100% and 110 mm/s.

**Fig. 1:**
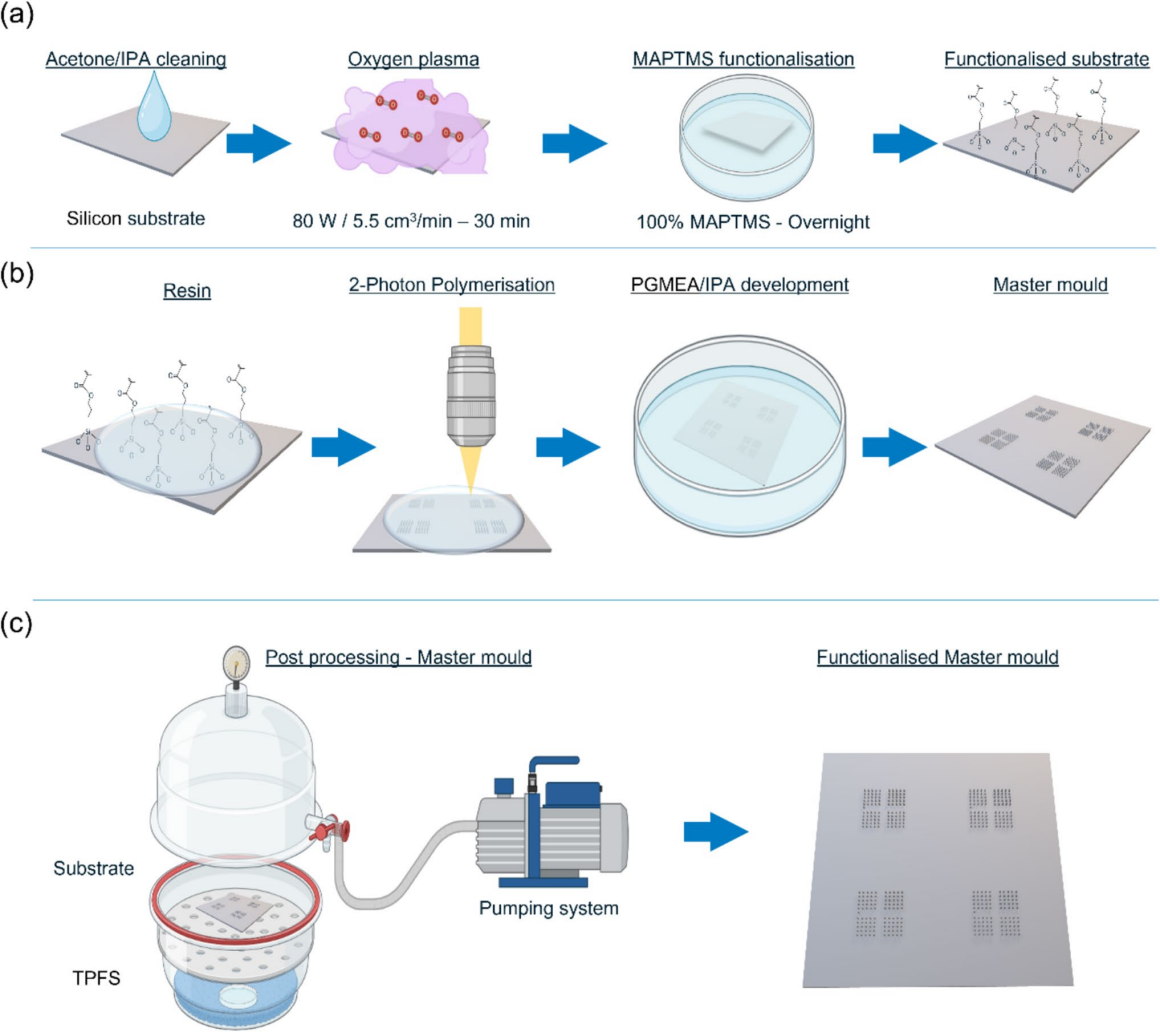
Schematical representation of the 2-photon polymerisation (2PP) and the trichloro (1H,1H,2H, 2H-perfluorooctyl) silane (TPFS) evaporation steps. (**a**) Before the 2PP step, the silicon substrate is cleaned with acetone and isopropyl alcohol (IPA) and undergoes oxygen treatment for better functionalisation with 3-(trimethoxy silyl)propyl methacrylate (MAPTMS) via the induction of silanol groups. (**b**) About 150/200 µl of resin is placed on top of the Si sample, and after the 2PP step, the non-polymerised resin is removed in a double-development step in PGMEA and IPA. (**c**) After drying, the master mould is functionalised with TPFS to prevent silicone adhesion.

The submillimetre-scale microneedle structures were printed on square silicon substrates. Before printing (Fig. 1a), the silicon substrates were first cleaned by rinsing with acetone and IPA, followed by activation in an oxygen plasma machine (Diener electronic GmbH + Co. KG) for 30 min at 80 W and an oxygen gas flow rate of 5.5 sccm. The substrate surface was covalently modified with methacrylate groups to improve the adhesion between the photo-polymerised resin and the silicon substrate. This silanisation was done by incubating the print side of the substrate with MAPTMS (100%) overnight. The samples were then rinsed with acetone and blown dry with an air gun. Afterwards (Fig. 1b), several drops of IP-Q resin were applied to the silanised silicon substrate. The printing process uses a layer-by-layer method and is monitored using a real-time camera. After the 2PP step, the samples were unloaded, and the remaining non-polymerised resin was developed in PGMEA for 7 min, followed by IPA for 3 min. Finally, the 2PP printed samples were dried with pressurised air before functionalisation.

### Microneedle Master Template Functionalisation

As the 2PP printed master mould material is not biocompatible and cannot contain biomacromolecules while retaining their functionality, another replication step is required to fabricate microneedles suitable for human skin application. To this end, inverse PDMS moulds were fabricated using the 2PP printed templates. Because PDMS can infiltrate and adhere to the 3D-printed surfaces, it is generally challenging to effectively demould the inverse silicone mould from the microneedle master after polymerisation of the PDMS. Therefore, a hydrophobic coating onto the acrylic master print was implemented to facilitate the demoulding (Fig. 1c). To this end, trichloro (1H,1H,2H, 2H-perfluorooctyl) silane (TPFS) was deposited onto the 2PP printed master from the gas phase [27]. This silanisation process was performed in a desiccator by adding 500 µl TPFS solution (100%) in a petri dish, on top of which the master mould was placed upside down in a holder made by fused deposition modelling (FDM) 3D printing (Prusa MK3S +, Prusa Research a.s., Prague, Czech Republic). A vacuum (1000 mbar) was applied overnight to complete the vapour deposition process.

### Replication by PDMS Moulding

**Fig. 2:**
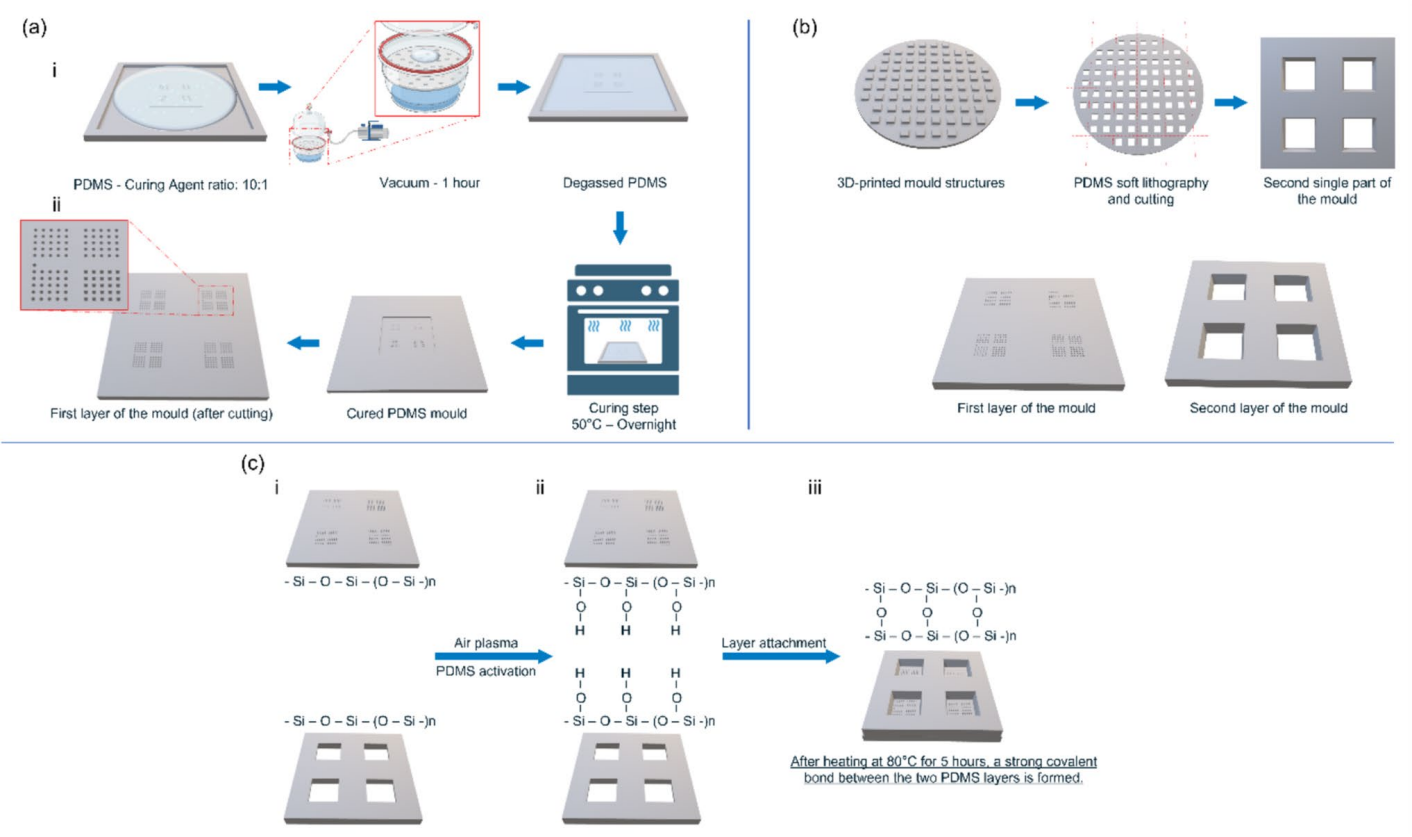
Representation of polydimethylsiloxane (PDMS) moulding and assembly. (**a**) PDMS moulding of the master mould. **i** PDMS solution is first poured and degassed, and (**ii**) after a heating step, it is solidified. (**b**) The second part of the final mould is moulded starting from a 3D-printed trapezoidal prism-shaped backplate. (**c**) (**i**) Air plasma-activated surfaces that (**ii**) once undergo the compressing and (**iii**) heating step, form a strong covalent bond.

PDMS was chosen as a material to produce the inverse microneedle moulds from the master prints (Fig. 2a). The PDMS base and the curing agent were mixed in a 1:10 ratio. Subsequently, this mixture was degassed in a desiccator for 15 min. Next, the mixture was poured into a container with the master mould and was degassed for another 30 min. These degassing steps are helpful to avoid the formation of air bubbles during the curing step. Subsequently, the container was placed in an oven (Memmert GmbH + CO. KG, Schwalbach, Germany) at 50°C, where the PDMS was cured overnight. Before demoulding, the PDMS was allowed to cool down to room temperature. IPA was infiltrated between the 2PP print and the PDMS mould to facilitate the peel-off, as it reduces the surface energy between the two.

Next, a second PDMS structure was fabricated, which was required to form the backplate of the microneedle arrays (Fig. 2b). To do so, an inverse template with the backplate structures was manufactured by FDM-3D printing. A squared trapezoidal prism with an upper edge of 6.5 mm, height of 2.5 mm, and angle of 10° was chosen for the backplate of the microneedle array to improve the demoulding step of the final polymeric microneedles. Once these second structures were cured, the pieces were cut singularly and bonded with the PDMS elements containing the microneedle moulds (Fig. 2c).

Before bonding, the two PDMS parts were cleaned with IPA in a Branson Ultrasonics CPX-952-536R cleaning bath (Branson Ultrasonics B.V., Ede, the Netherlands) for 5 min and blown dry with air. Both PDMS elements were placed on top of a first square holder made of polymethyl methacrylate (PMMA), and their surfaces were activated by air plasma with a PiezoBrush PZ3 plasma gun (Relyon plasma GmbH, Regensburg, Germany) for 2 min (1 min each). With the help of vacuum tweezers (RDPRO from RS Components B.V., Haarlem, the Netherlands), the PDMS layer containing the backplate design was aligned and placed on top of the PDMS layer containing the microneedle negatives, which was still resting in the PMMA holder. Eventually, a second PMMA holder was placed on top of the two-centred PDMS layers. The two PMMA holders were held in place using two clamps to avoid uneven deformation of the PDMS moulds. Finally, the clamped assembly was placed in an oven for 5 h at 80°C to allow covalent bonding of the two PDMS elements. After that, the clamps and PMMA holders were removed, and the moulds were stored at room temperature until use.

### Fabrication of Polymeric Microneedle Arrays

To screen the microneedle design parameters that affect human skin piercing, polymeric microneedle arrays were fabricated utilising PVA (7.5% w/v), a biodegradable polymer. PVA was dissolved in PBS that contained 100 µg/mL sodium carboxy-fluorescein (facilitating fluorescence microscopic analysis) under vigorous vortexing. To fill the cavities of the inverse PDMS microneedle mould, 50 µL of the polymer formulation was pipetted into the moulds and centrifuged with the Rotanta 460R centrifuge (Hettich, Kirchlengern, Germany) at 3750 RPM, 35°C for 1 h. After centrifugation, the moulds were vacuum (340 mbar) dried (Binder, Tuttlingen, Germany) at 35°C overnight. To obtain a solid microneedle support, a backplate layer of epoxy glue was added to each array and dried (35°C) overnight. The epoxy glue facilitates the demoulding of polymeric microneedle arrays from the inverse PDMS moulds and forms a solid support for handling. After microneedle array removal, the moulds were inspected for potential incomplete demoulding by microscopy, checking the presence of fluorescent residuals. Polymeric microneedle arrays were stored under vacuum (340 mbar) until use.

### Characterisation of Master Templates and Polymeric Microneedle Arrays

For visual characterisation, the master template and the polymer microneedles were imaged using a fluorescence stereomicroscope (Leica Biosystems, Amsterdam, the Netherlands) equipped with a GFP filter set (excitation wavelength: 460–90 nm/emission wavelength: 510 nm).

### Microneedle Design Parameters

This study systematically investigated microneedle design parameters to determine their effect on human skin piercing. With the developed general workflow for microneedle fabrication (Figs. 1 and 2), a microneedle toolbox was designed (Table I). Based on a combination of prior literature and practical design consideration the different microneedle design parameters were selected, including: microneedle geometry (cones and obelisks), base shape (circular, tetragonal, hexagonal, and octagonal), base diameter (50—200 µm), tip diameter (3—50 µm), length (250—650 µm) and microneedle density (400—2304 cm^−2^). All microneedle design parameters were labelled with alphabet letters (A-D) incorporated on each array for tracking and identification. Each template is designed to be a 5 mm × 5 mm square area (30 µm/thick) and contains 101 needles, 25 for each base shape, and one "reference microneedle" for traceability and parameter orientation. These microneedle array designs allowed the analysis of four different designs during a single application on *ex vivo* skin.

**Table I:**
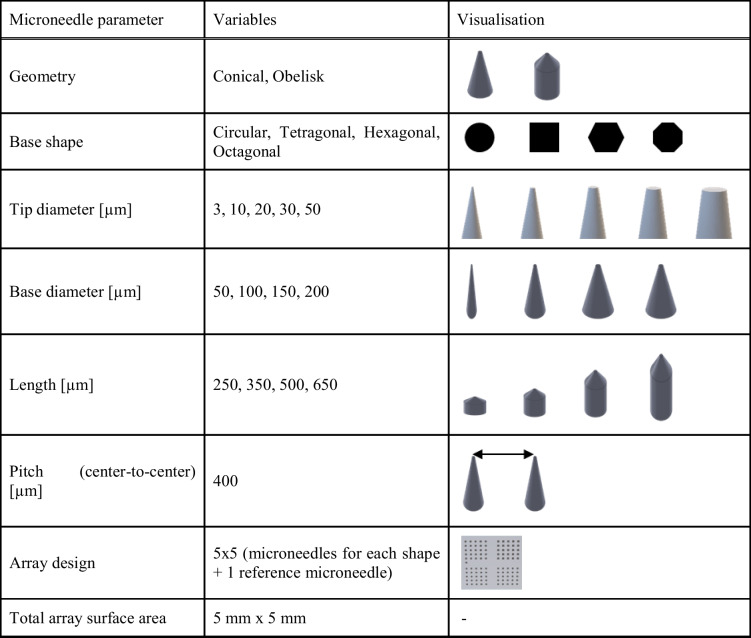

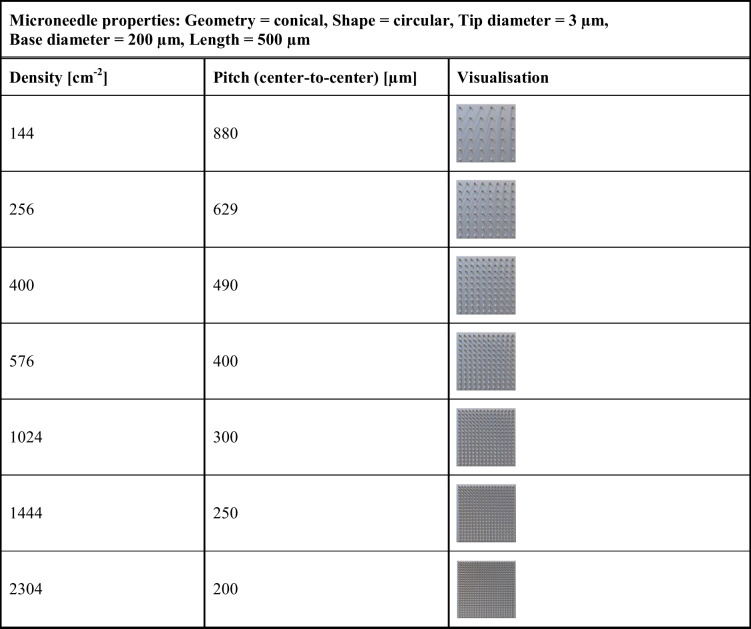
Master Microneedle Template Dimensions and Design Parameters

### Evaluation of Microneedle Design Parameters on Human Skin Piercing

To understand how microneedle design parameters influence their efficacy in piercing skin, the previously described microneedle arrays were applied onto *ex vivo* human skin. This study assessed the nuanced relationship between microneedle geometry parameters and skin penetration. The approaches that were performed are described in detail below.

### Human Skin

The repeatability and accuracy of the microneedle applications were evaluated in full-thickness abdomen *ex vivo* human skin (obtained from local hospitals after written informed consent from the donors and handled according to the Declaration of Helsinki Principles) [27]. Excess fat was removed, and the skin was stored at −80°C until use. Before the microneedle application studies, the skin was thawed at 37°C for one hour in a petri dish containing wet tissues to prevent dehydration. Subsequently, the skin was stretched on parafilm-covered Styrofoam and cleaned with 70% ethanol and milli-Q water.

### Application of Polymeric Microneedle Arrays onto *Ex Vivo* Human Skin

Ex vivo human skin penetration tests were performed to determine the piercing efficiency of the different dMNA designs and geometries. A digitally controlled impact-insertion applicator (uPATCH B.V., Leiden, the Netherlands) was used to pierce the skin with an impact velocity of 0.4—1.4 m/s [28]. All polymeric microneedle arrays were mounted onto the microneedle mount of the applicator using double-sided adhesive tape (Tesa, Hamburg, Germany). Each microneedle array was applied once on the *ex vivo* human skin and was automatically retracted from the skin after 1.0 s.

### Determination of Piercing Efficiency by Trypan Blue Assays

To assess skin piercing, 70 µL of 0.04% trypan blue was applied onto the microneedle-treated skin site. After 45 min, the excess trypan blue solution was removed. To prevent overestimating the penetration efficiency (potential formation of indentations after microneedle application), the stratum corneum was removed by tape-stripping in alternating directions until the skin appeared shiny (approximately ten times). Finally, the skin was analysed by microscopy to determine the number of blue spots, indicating the number of penetrated microneedles in the skin. Penetration efficiency was determined by dividing the number of penetrations by 25, the total number of the same microneedles in one array. Penetration studies were carried out in triplicate for each microneedle design.

### Statistics

Statistical analysis was performed using GraphPad Prism version 8.4.1 (La Jolla, CA, USA). Data was tested using Kruskal–Wallis and Mann–Whitney tests. p < 0.05 was considered significantly different.

## Results and Discussion

The main objective of this study was to generate a workflow for the fabrication of solid microneedles. Utilising 2PP and PDMS mould fabrication, a general workflow for microneedle fabrication was developed and optimised, which resulted in a reproducible and traceable process for polymeric microneedle array fabrication. With this workflow, a microneedle toolbox was created that contains a multitude of microneedle designs, which allows studying the effect of a single microneedle design parameter on skin piercing. To determine the critical microneedle design parameter for skin piercing, *ex vivo* human skin was used.

### Master Template Fabrication

**Fig. 3:**
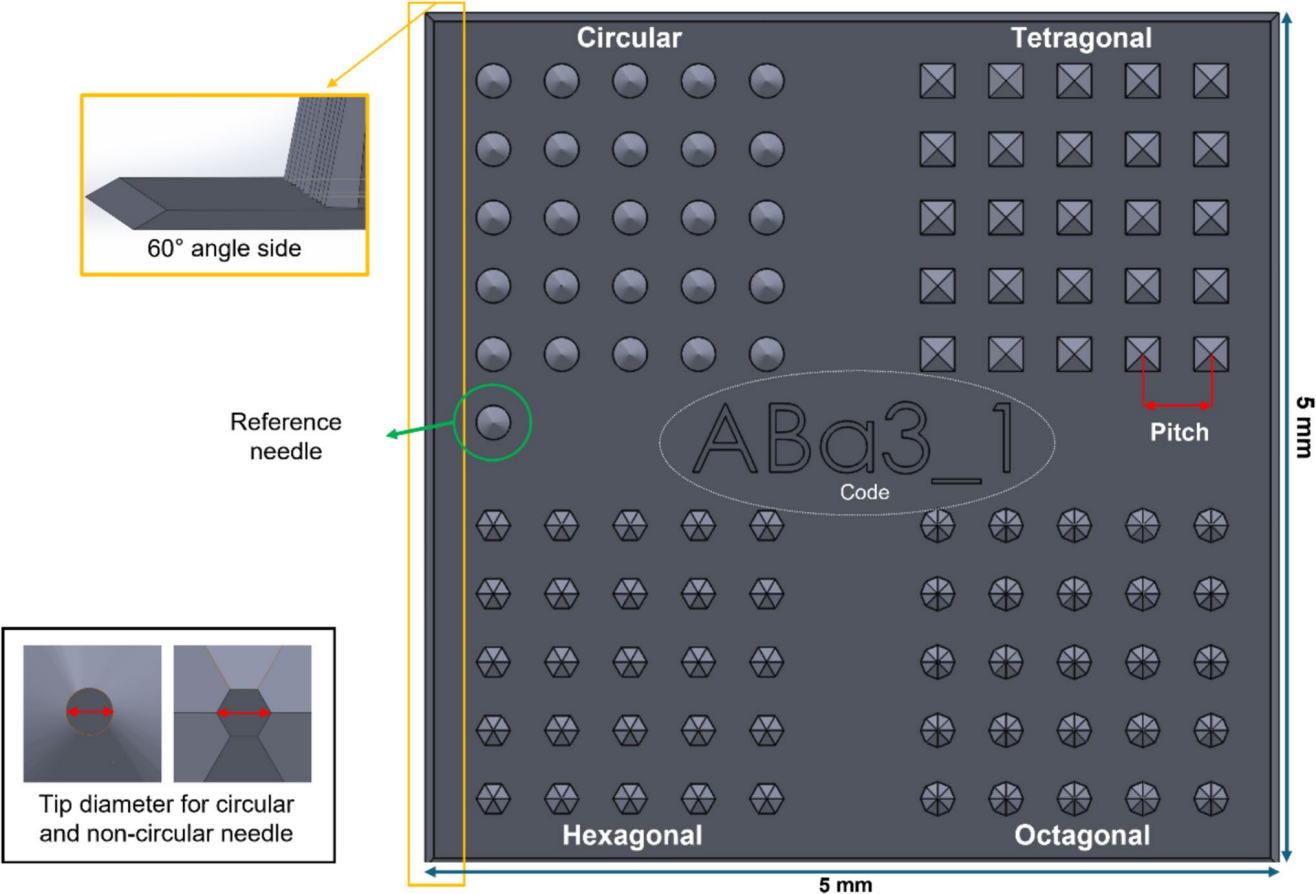
Master microneedle template dimensions and design

The master templates were designed to allow simultaneous investigation of four different microneedle shapes upon application to the skin. Each template consists of four distinct areas, each containing 25 microneedles with either circular, tetragonal, hexagonal, or octagonal shapes. Additionally, all microneedle arrays include a reference microneedle and a coding feature to serve as a point of orientation (Fig. 3).

The master template CADs for the various microneedle design parameters were successfully developed using SolidWorks software. More than 75 different designs were fabricated into master templates, whereby one design parameter varied at the time, making it possible to evaluate individual parameters. These parameters are important when continuing towards intradermal delivery and/or sampling. For example, microneedle length influences how deep the microneedles penetrate the skin. Too-short microneedles may not penetrate the SC, while too-long microneedles may reach beyond the optimal layer within the skin for the desired immunological responses or reach pain receptors and blood vessels. On the other hand, the microneedle tip diameter is associated with the microneedles' sharpness and the total surface, which may increase friction and thereby increase insertion forces. Furthermore, by increasing the tip diameter and density, a larger skin area can be covered, targeting more immune cells for drug/vaccine uptake, which could be beneficial for immune therapeutics (*i.e.*, reaching more DCs) [17]. The master template CAD ensures uniformity and accuracy in microneedle template production through meticulous detailing and adherence to the design parameters (Table I).

**Fig. 4:**
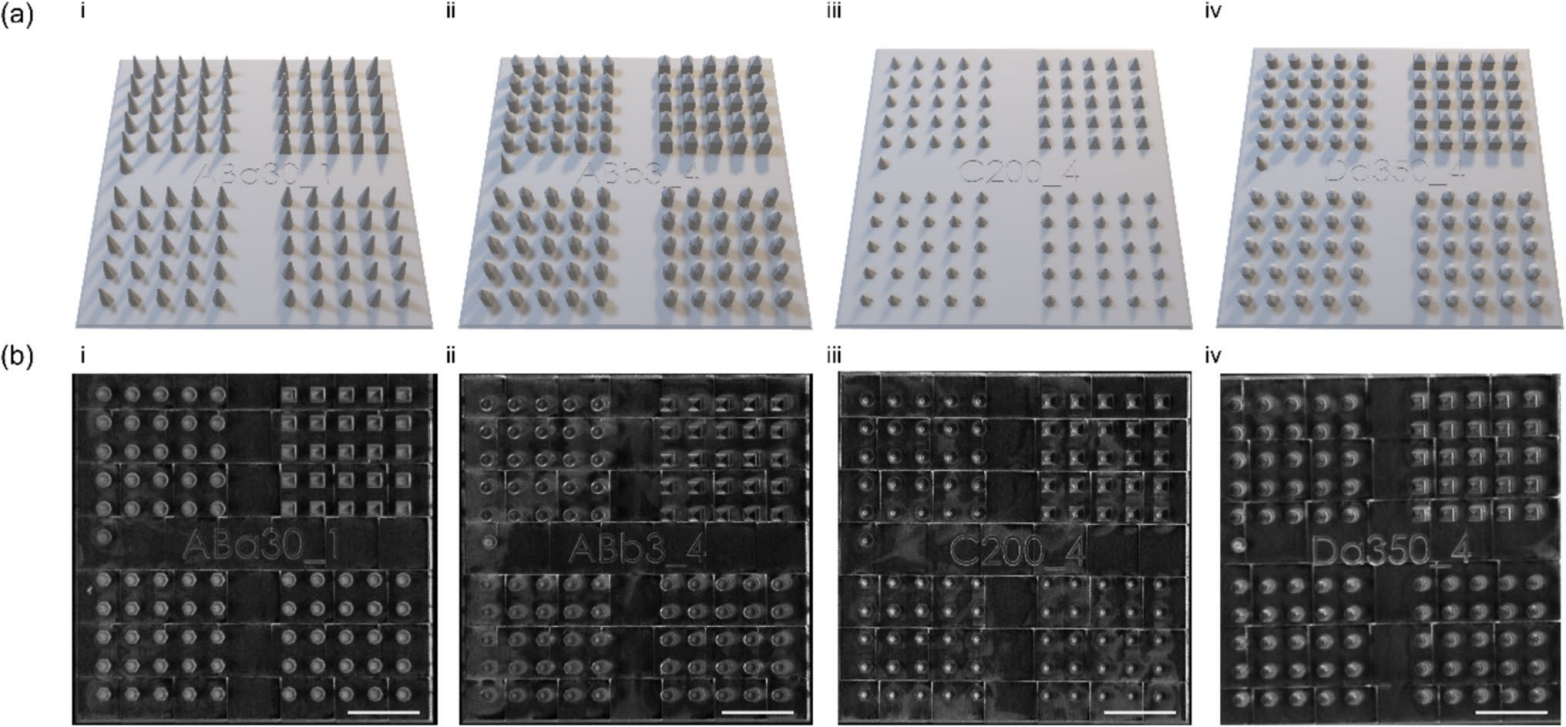
Master microneedle template and design parameters. (**a**) Master template designs for solid microneedle arrays created with the SolidWorks software. (**b**) Stereomicroscopic images of 2PP master template of different designs. **i** Master structure of conical microneedle arrays with tip diameter of 30 µm, pitch distance of 400 µm, length of 500 µm and microneedle diameter of 200 µm. **ii** Master structure of obelisk microneedle arrays with tip diameter 3 µm (pitch distance of 400 µm, length of 500 µm and microneedle diameter of 200 µm. **iii** Master structure of conical microneedle arrays with a needle diameter of 200 µm, pitch distance of 400 µm, length of 250 µm and tip diameter of 3 µm. **iv** Master structure of obelisk microneedle arrays with variations in microneedle length of 350 µm, pitch distance of 400 µm, tip diameter of 3 µm and microneedle diameter of 200 µm. Scale bar = 1 mm.

Regarding the various designs, the reference microneedle always had a circular base shape, conical geometry, 3 µm tip diameter, and 200 µm base diameter, and the length was changed according to the sample length of the other microneedles in that specific 4 × 25 microneedle array design. This reference microneedle was used to identify and trace specific microneedle base shapes after skin application. To enable the determination of statistical differences in the piercing ability of four different microneedle designs during a single skin application (utilising only minimal amounts of human donor tissue), we designed our microneedle arrays in such a way that they contained 25 microneedles per design on 5 × 5 mm^2^. Furthermore, each template has been labelled with a specific code that was used for traceability from 2PP printing to dermal application (Fig. 4). These codes are also present on the polymeric microneedle arrays due to microreplication.

During initial optimisation studies, we could not remove the silicone inverse mould without disrupting the microneedles from the master acrylic template. Therefore, we optimised the master template design by adding a base below the printed microneedles. The base increases the surface area of the printed structures with the silicon substrate to withstand the forces applied during the demoulding of the inverse microneedle moulds. By placing a base plate 30 µm-thick and 60° drafted sides, the applied forces on the single projections/needles are reduced, improving the efficiency of the demoulding step and increasing the number of (re)uses of the master moulds.

The designs were imported to the Nanoscribe software for 2PP processing and template fabrication. Visual characterisation demonstrated that after processing, the master templates correspond to their SolidWorks design and represent 101 intact needles, including one reference microneedle for orientation purposes (Fig. 4b). The small-scale production of these master templates using 2PP printing proved to be highly efficient. However, the limited printing speed and small build volume may pose challenges for scaling up to larger or high-throughput designs. Nonetheless, alternative approaches that can be implemented in our methodology, for example quartz mould fabrication, however, these technologies are quite expensive compared to 2PP [29]. The IP-Q resin is biocompatible (no cytotoxic effect, according to the supplier) after polymerisation, and their residues are minimal or absent in subsequent master template printing. However, for translational purposes and further minimizing potential IP-Q residues in the PDMS mould, 2PP printing should be followed by vigorous rinsing and cleaning to remove leachable and unreacted materials.

### Inverse Silicone Mould Fabrication

The inverse silicone microneedle moulds were fabricated from PDMS. Because this silicone is transparent, gas-permeable, and conforms to the surfaces it comes into contact with. Furthermore, PDMS is available as a medical-grade elastomer, which is widely used in medicine and high-tech devices (*i.e.*, medical implants), enabling the usage of these inverse silicone PDMS microneedle moulds for future GMP production [30]. Therefore, PDMS is a well-suited material for making inverse replicates of the acrylic microneedle master templates. Additionally, a second PDMS mould was made for the generation of a strong backplate for the microneedles.

**Fig. 5:**
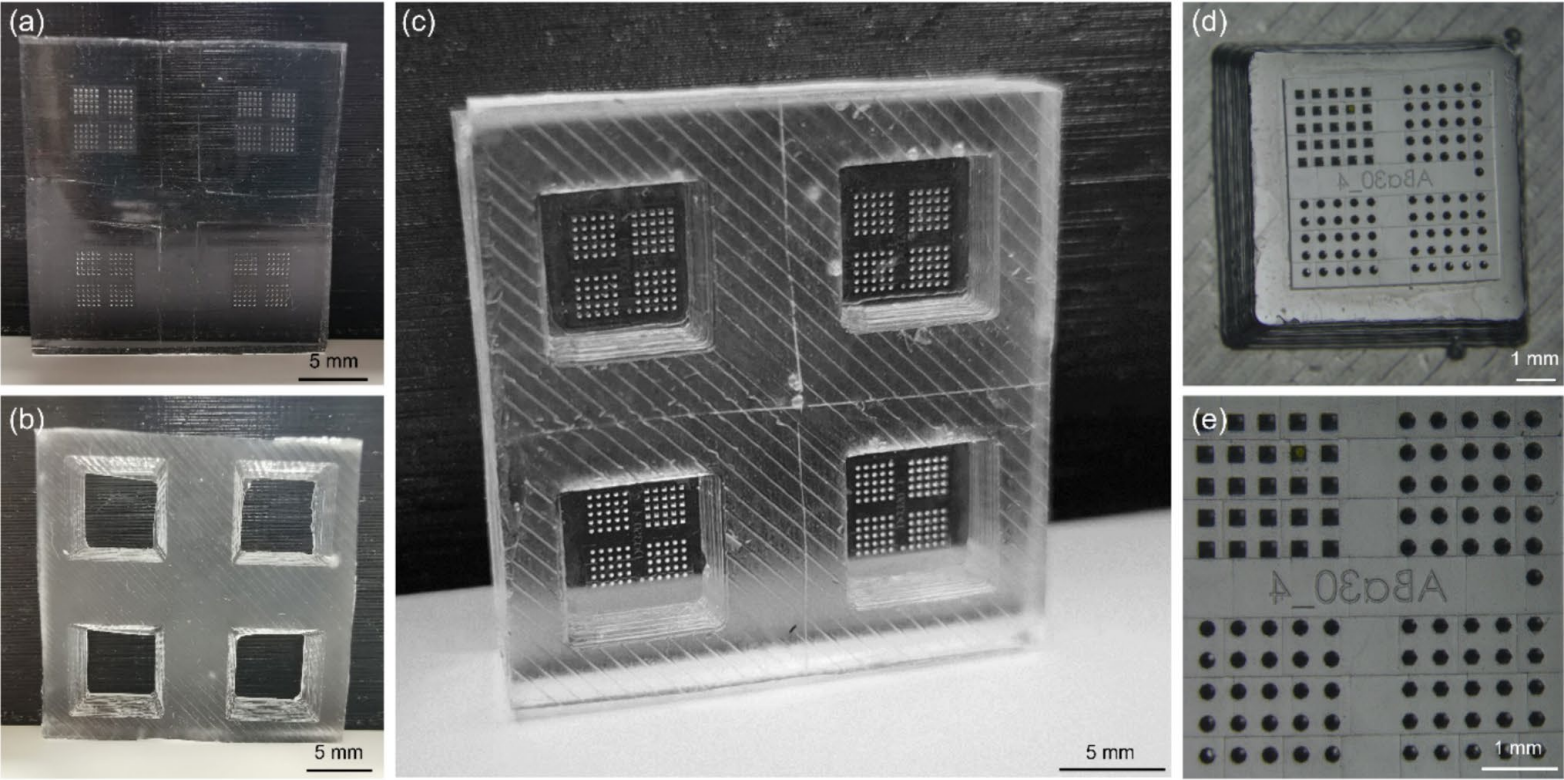
(**a**) Inverse polydimethylsiloxane (PDMS) replicate of the acrylic microneedle master template. (**b**) PDMS mould for the generation of a microneedle backplate. (**c**) Complete inverse PDMS microneedle mould. (**d**) Stereomicroscopic images of single microneedle array PDMS mould’s detail. (**e**) Stereomicroscopic image template’s detail.

To reproducibly attach the two separate PDMS layers, plasma activation was combined with applying force and heat to bond the two surfaces (Fig. 5). Plasma removes the organic material at the surface and creates silanol surface groups (SiOH) that make the PDMS surface hydrophilic. Strong covalent bonds, Si–O-Si, are formed once the activated PDMS comes into contact with another activated PDMS surface. However, applying plasma to the same area of the microneedle cavities interferes with the polymer formulations utilised to produce the final microneedles. This resulted in non-detachable polymeric microneedles in the PDMS mould after solidification (data not shown). For this reason, a 3D-printed shadow mask was created that covered the microneedle template area. The mask allowed the activation of the surface required to bond the mould for the backplate while protecting the inverse microneedle templates. For clinical translational, medical-grade PDMS is available for inverse microneedle mould production.

### Polymeric Microneedle Array Fabrication

PVA (7.5% (w/v)) was selected as the polymer for microneedle array fabrication due to its relatively high mechanical strength and a slow dissolution rate compared to other water-soluble polymers commonly used in microneedle fabrication, such as polyvinylpyrrolidone (PVP). These properties minimised the risk of microneedle damage or deformation during demoulding and handling, thereby preserving the structural integrity of the fabricated arrays. It is important to note, however, that other polymers, such as PVP and hyaluronic acid, are also compatible with the described fabrication workflow (data not shown). The choice of polymer should be tailored to the specific (therapeutic) application, optimising microneedle formulations accordingly. The current materials used for polymeric microneedle array production are all GMP-compliant materials. However, for translation towards the clinic, fabrication should be performed under strictly controlled conditions.

**Fig. 6:**
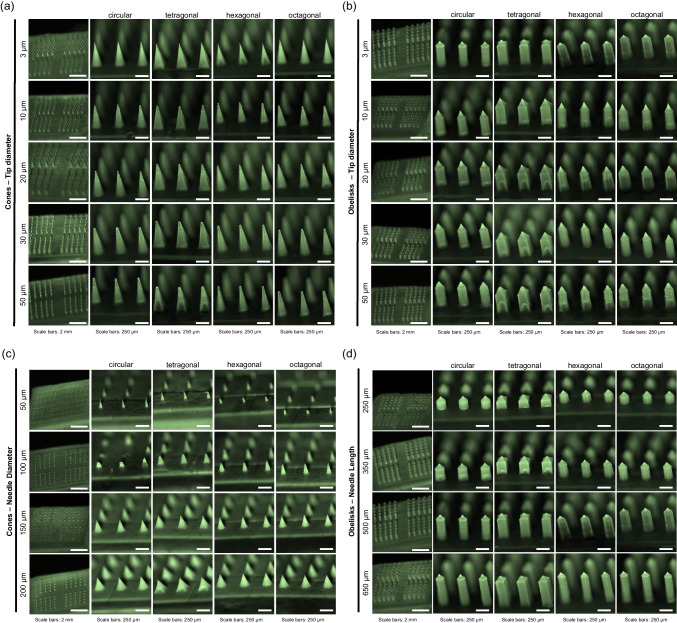
Stereomicroscopic fluorescence images of polymeric microneedle arrays with different geometries (cones and obelisks) and shapes. All microneedle arrays (5 × 5 mm) include 25 microneedles of each shape (circular, tetragonal, hexagonal and octagonal), resulting in a total of 101 needles (including a reference microneedle). Microneedle arrays were imaged from a lateral view with an 80-degree angle. (**a**) Conical microneedle arrays with variations in tip diameter (3, 10, 20, 30 and 50 µm) (pitch distance of 400 µm, length of 500 µm and microneedle diameter of 200 µm). (**b**) Obelisk microneedle arrays with variations in tip diameter (3, 10, 20, 30 and 50 µm) (pitch distance of 400 µm, length of 500 µm and microneedle-diameter of 200 µm). (**c**) Conical microneedle arrays with variations in diameter (50, 100, 150 and 200 µm) (pitch distance of 400 µm, length of 250 µm and tip diameter of 3 µm). (**d**) Obelisk microneedle arrays with variations in microneedle length (250, 350, 500 and 650 µm) (pitch distance of 400 µm, tip diameter of 3 µm and microneedle diameter of 200 µm).

To visualise the different geometries and shapes of the different microneedle designs, fluorescence stereomicroscopy imaging was performed (Fig. 6), and the dimensions of the polymeric microneedle arrays were determined (Supplementary data). All the arrays were effectively removed from the inverse PDMS moulds without leaving residues behind within the mould (Supplementary Fig. 1), as determined by inspection of the moulds by fluorescence microscopy. Microscopic evaluation of the conical- (Fig. 6a) and obelisk- (Fig. 6b) geometries revealed intact microneedles with varying tip diameters (base diameter of 200 µm, length of 500 µm). Additionally, the diameter of the microneedle base was changed among the conical microneedle geometries as a variable (Fig. 6c). Image analysis showed that, with the current methodology, intact and sharp microneedles can be made with a microneedle diameter of 150 µm and 200 µm (tip diameter of 3 µm, length of 250 µm). However, the designs with smaller microneedle diameters, 50 and 100 µm, resulted in fragile microneedles. Small-diameter needles have less material volume and structural support, making them more prone to production and application failures. These microneedles cannot withstand the forces encountered during demoulding, leading to ruptures and bent tips. Furthermore, the overall design of the microneedles, including the aspect ratio, plays a critical role in their integrity [23]. One solution to increase the success of generating intact microneedles with lower base diameters is to optimise the fabrication process for these designs, utilising other polymers/materials [31, 32]. PVA (7.5% (w/v)) used for these designs may not possess the optimal balance of stiffness and flexibility needed for such structures. In contrast, other types of solid microneedles (*i.e.*, silicon, metal, and ceramic) or other polymers might succeed in the fabrication of microneedle arrays with small microneedle diameters (< 100 µm) [33, 34]. PDMS moulds have been used in previous studies for the fabrication of several polymeric arrays [33, 34], but also the fabrication of ceramic [35], titanium and stainless-steel [36] microneedles. This suggests that our generated microneedle fabrication procedure can be followed for microneedle production from other materials.

Finally, obelisk microneedles with different lengths (250, 350, 500 and 650 µm) were designed and fabricated (Fig. 6d) (tip diameter of 3 µm, base diameter of 200 µm), resulting in intact polymeric arrays for all four designs regarding length. Microscopic evaluations indicate that almost all designs, except microneedles with 50 µm and 100 µm base diameters, could be made with the current methodology and selected polymer. The stereomicroscopic images were used to determine actual microneedle dimensions (total, shaft, tip length, and base and tip diameter) (Supplementary Table I—IV). These measurements showed that the polymeric microneedle arrays are replicates of their corresponding CADs. The microneedles’ geometry (conical and obelisk) and shape (circular, tetragonal, hexagonal and octagonal) were confirmed. Regarding the microneedle design parameters, length and diameter, polymeric microneedles had an average compatibility with the expected CAD-based sizes of 92%. A slight size reduction was found between the polymeric microneedles and the CAD-based sizes: 11% for length, 14% for base diameter and 2% for tip diameter. Several factors can cause these minor differences. One explanation is the imaging angle during microscopic evaluation, which could have led to slight distortions in the dimensions. Additionally, minor inaccuracies in the stereomicroscope setup (*i.e.*, device calibration or limited resolution) could contribute to the value differences observed in the dimensions with respect to the CADs. Furthermore, during the production process, particularly during curing, the microneedles may undergo shrinkage; any variations in temperature, pressure, or material behaviour during moulding and polymerisation could contribute to reductions in size compared to the original CADs.

### Application of Microneedle Arrays onto *Ex Vivo* Human Skin

**Fig. 7:**
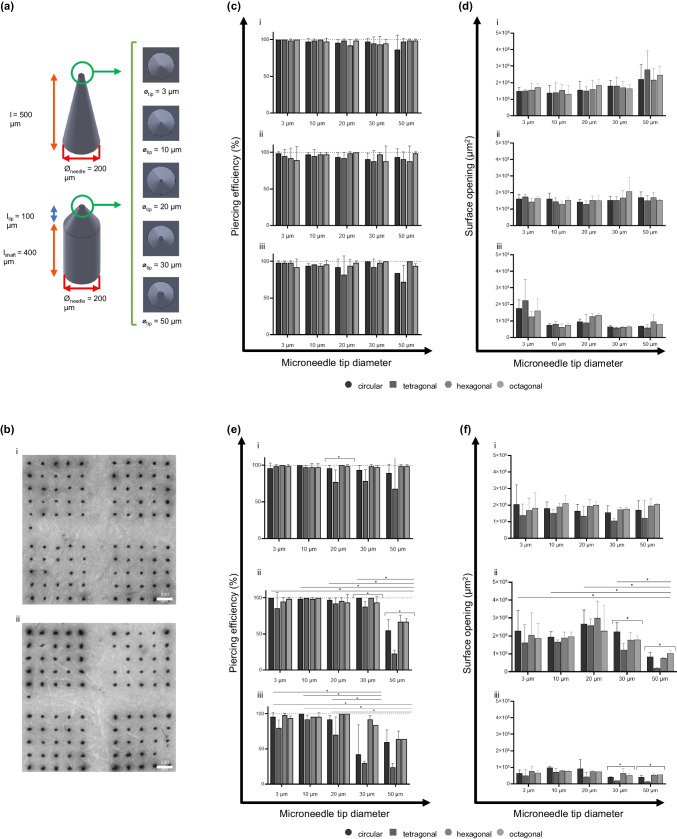
Piercing efficiency of polymeric microneedle arrays with varying microneedle tip diameter parameters. Schematic representation of the microneedle arrays (**a**) designs representing the conical and obelisk microneedles with varying tip diameters. (**b**) Representative micrograph of trypan blue assays on *ex vivo* human skin to determine piercing efficiency for the conical (**i**) and obelisks (**ii**) arrays. (**c**-**d**) Piercing efficiency for the conical microneedle arrays with different shapes (circular, tetragonal, hexagonal and octagonal) and tip diameter: (**i**) impact velocity was 1.4 m/s, (**ii**) impact velocity was 1.0 m/s, and (**iii**) impact velocity was 0.4 m/s. (**e**–**f**) Piercing efficiency for the obelisk microneedle arrays with different shapes (circular, tetragonal, hexagonal and octagonal) and tip diameter: (**i**) impact velocity was 1.4 m/s, (**ii**) impact velocity was 1.0 m/s, and (**iii**) impact velocity as 0.4 m/s.

**Fig. 8:**
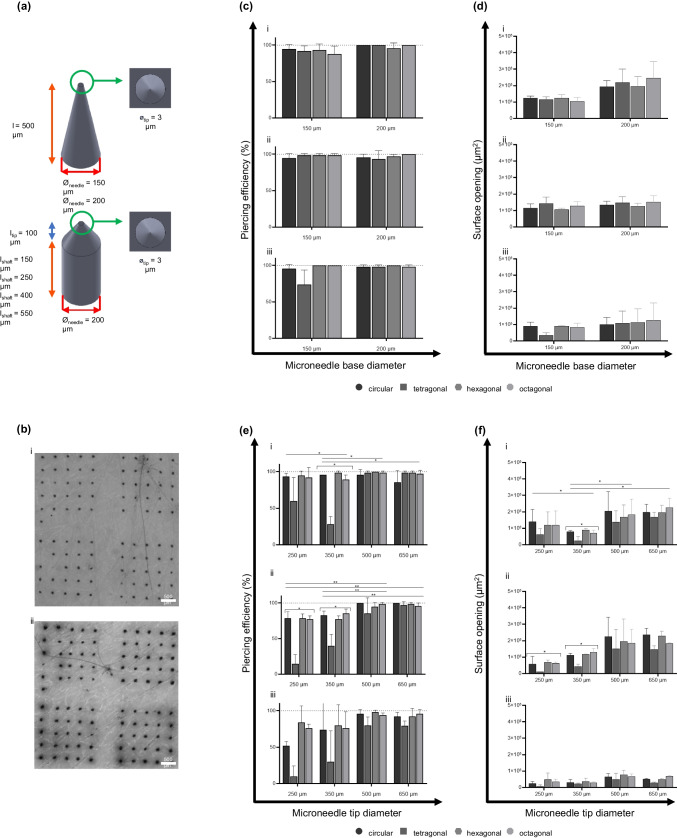
Piercing efficiency of polymeric microneedle arrays with varying microneedle-base diameter and length parameters. Schematic representation of the microneedle arrays (**a**) designs representing the conical microneedles with varying base diameter and obelisk microneedles with varying length. (**b**) Representative micrographs of trypan blue assays on ex vivo human skin to determine piercing efficiency for the conical (**i**) and obelisks (**ii**) arrays. (**c**-**d**) Piercing efficiency for the conical microneedle arrays with different shapes (circular, tetragonal, hexagonal and octagonal) and base diameter: (**i**) impact velocity of 1.4 m/s, (**ii**) impact velocity of 1.0 m/s, and (**iii**) impact velocity of 0.4 m/s. (**e**–**f**) Piercing efficiency for the obelisk microneedle arrays with different shapes (circular, tetragonal, hexagonal and octagonal) and microneedle length: (**i**) impact velocity of 1.4 m/s, (**ii**) impact velocity of 1.0 m/s, and (**iii**) impact velocity of 0.4 m/s.

Intact polymeric microneedle arrays were applied onto *ex vivo* human skin explants to determine the piercing efficiency (Figs. 7 and 8). These piercing tests are based on impact speed, addressing a critical and ongoing question in the field regarding how puncture strength and impact speeds influence penetration (and drug delivery) efficiency. Common approaches in the literature often rely on thumb-pressure application or force-based applicators, which can introduce variability and inconsistency due to differences in applied force between users. The velocity-controlled application provides a systematic and reproducible method to evaluate piercing efficiency [28]. Compared to other skin models, such as pig or mice skin or synthetic gels, human skin explants offer a unique advantage in mimicking the complexity and behaviour of human skin, providing a more accurate representation of *in vivo* conditions compared to alternative skin models [37, 38]. Moreover, human skin explants maintain the intricate cell biological and biomechanical properties inherent to human skin, including thickness, composition variations, and nonlinear viscoelasticity [39]. When microneedles are applied to the skin, they cause deformation of the skin surface [40]. Therefore, microneedle design and insertion force/velocity are essential for microneedle piercing efficiency [41]. An applicator can facilitate efficient and controlled microneedle insertion into the skin. Using an impact-insertion applicator allows for improved efficiency and reproducibility of microneedle array insertion into the skin compared to pressing force-based and (uncontrolled) manual applications [28, 42]. Thus, microneedles should hold down substantial insertion forces without causing pain and experiencing design deformations.

Our data showed that almost all selected microneedle designs (Fig. 7a and 8a) displayed excellent penetration efficiencies in human abdominal skin (Fig. 7c**/e-i** and **8c/e-i**) by an impact power of 100%, corresponding to a velocity of approximately 1.4 m/s. To investigate whether design parameters affect skin application, the impact velocity was reduced to 75% and 50% (1.0 and 0.4 m/s, respectively). For conical microneedles with varying tip diameters (Fig. 7c), 55 of 60 arrays displayed > 90% penetration efficiency regardless of impact velocity. The lowest penetration efficiencies were shown for the tetragonal-shaped microneedles with a tip diameter of 20 µm or higher (70—85%) (not significant). Previous studies determined the penetration depth of conical microneedles with tip diameters ranging from 5 to 37 µm [43]. The authors reported that microneedles with diameters less than 15 µm are essential to insert microneedles in a well-controlled manner to a desired depth. Another study used computational modelling to investigate tip sharpness and the perception of pain after skin insertion. Here, they indicated that the tip diameters influence both the mechanical properties and the pain induction associated with microneedle application. A tip diameter ranging from 1 to 25 µm has been associated with a painless, but efficient, insertion. For lengths above 1000 µm, the microneedles tend to gain frictional resistance, resulting in pain induction [44]. The present study found no difference in piercing efficiency between the different tip diameters.

The same experiments were performed for the obelisk microneedles with variation in tip diameter (Fig. 7e). As for the conical microneedles, the tetragonal-shaped designs (tip diameter < 20 µm) were demonstrated to be the least efficient (24—70%) in piercing *ex vivo* skin compared to the other shapes (p < 0.05). In parallel, the surface of the skin openings was measured after the microneedle array application (Fig. 7d-f). These graphs show that the surface of the skin opening (caused by the microneedles) decreased together with reduced impact velocity and increased tip diameter.

The piercing efficiency experiments were also performed for the arrays with a variable base diameter (Fig. 8a-i) and length (Fig. 8a-ii) design parameters. Similar to the previously described data for the other designs, the penetration tests (Fig. 8b) showed the highest piercing efficiency when arrays were applied with an impact velocity of 100% (Fig. 8c-i and 6e-i). The conical microneedles with varying base diameters show, regardless of impact velocity, high piercing efficiency with an average of 96% (Fig. 8c). No significant differences were observed between arrays with varying base diameters. The study of Kochhar *et al.* demonstrated that 300 and 400 µm base-diameter microneedles with an average length of 850 µm showed the most promising high-efficiency penetration profiles; in comparison, 200 µm base-diameter microneedles had a low penetration efficiency [45]. This might be attributed to the microneedle's thin base, resulting in a weaker structure that buckles more easily upon application onto the skin's surface than microneedles with larger base diameters. Besides piercing efficiency, the skin’s surface openings after microneedle array application (Fig. 8d) were demonstrated to be slightly affected by the reduced impact velocity; however, the differences were not significant.

Utilising obelisk-shaped microneedles demonstrated a reduced piercing ability upon dermal application when their length is below 500 µm (p < 0.05) (Fig. 8e). Similar reduced piercing efficiency has been reported in the literature, referring to increased transdermal drug delivery when using longer microneedles (> 600 µm) compared to arrays with short microneedle lengths (< 300 µm) [40, 46]. It was postulated that the skin's elasticity counteracted microneedles' penetration ability when inserted with a reduced impact velocity (1.0—0.4 m/s) into the skin. This might be a result of the skin’s elastic properties. The viscoelasticity of the skin could cause deformations of the skin after microneedle application [39, 40].

Despite increasing the applicator impact velocity can partially circumvent deformation, independent of the length, the tetragonal microneedles showed a significant reduction in their ability to pierce human skin (*i.e.*, average piercing efficiency for length 250 µm was 27% and length 350 µm was 37%). Regarding the surface openings after microneedle application for these designs, as for the tip diameter variants, the graph shows that the skin opening decreased together with a reduced impact velocity. However, the surface of the opening increased when the microneedles increased in length (Fig. 8f). This might be due to deeper penetration of the longer microneedles, where they create larger and possibly wider openings [47].

Among all design parameters, microneedles with a circular shape showed the highest piercing efficiencies. In contrast, the arrays with the tetragonal needles significantly displayed the lowest efficiency in skin piercing. Cordeiro *et al.* showed that circular-shaped microneedles have a better insertion capability at lower impact velocities than other shapes (tetragonal, cross-shaped, and pedestal). This can be explained by the circular microneedles' minimised surface area. Circular microneedles have a smaller contact surface area at their tip, which allows for a more concentrated application force. This concentration of force makes it likely easier to penetrate the skin, which has relatively tough and elastic characteristics [39, 40].

### Effect of Microneedle Density on Skin Piercing

The microneedle density is a critical factor that might influence microneedle piercing into human skin upon application. The 'bed-of-nails' effect has been reported to potentially influence skin piercing [48–50]. For example, Loizidou *et al.* demonstrated enhanced drug flux for microneedle arrays containing tetragonal needles with a length of 400 µm and 2000 needles/cm^2^ in needle density; in contrast, increased microneedle density (5625 needles/cm^2^) had a lower drug flux [51]. Beyond piercing efficiency, microneedle density can also affect therapeutic outcomes. Coffey *et al.* investigated how increasing microneedle array density from 5.000 to 30.000 needles per cm^2^ enhances epidermal cell death and boosts antibody responses to influence vaccination, demonstrating a direct adjuvant effect of density on immune activation [52]. However, it should be noted that microneedle density is not the only parameter that might influence therapeutic outcomes [53–55]. Crichton *et al.* investigated a microneedle array containing fewer, flat-shaped microneedles, with improved increasing depth and greater localised skin cell death and therefore immune infiltration. They tested their array with an Influenza vaccine, generating a comparable immune response to traditional injections using 1000 times less vaccine dose [56].

**Fig. 9:**
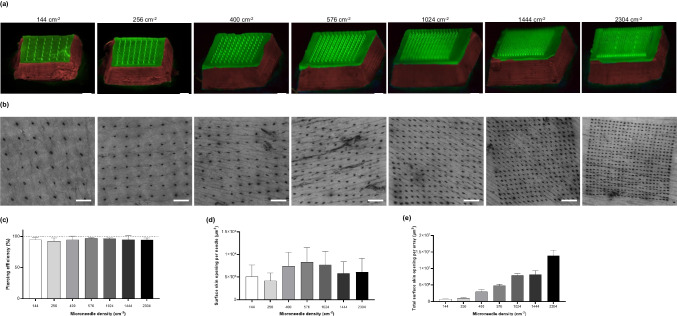
Polymeric microneedle arrays with different microneedle densities. (**a**) Representative stereomicroscopic fluorescence images of microneedle arrays with different densities. All microneedle arrays (5 × 5 mm) consist of conical microneedles with a circular shape (length of 500 µm, tip diameter of 3 µm, base diameter of 200 µm). Microneedle arrays were imaged from a lateral view with an 80-degree angle. (**b**) Trypan blue assays on *ex vivo* human skin to determine piercing efficiency for each density microneedle array. (**c**) Piercing efficiency for the different microneedle arrays applied on the skin with an impact velocity of 1.4 m/s. (**d**) The surface of the skin opening per microneedle after patch application. (**e**) The total surface of the skin opening per array after microneedle application. Scale bar = 1 mm.

With the developed methodology, the spacing and arrangement of the microneedles were carefully controlled, fabricating microneedle master structures containing conical microneedles with a circular shape (length of 500 µm, tip diameter of 3 µm, and a base-diameter of 200 µm), with densities varying between 144 and 2304 cm^−2^. Polymeric microneedle arrays were fabricated following the optimised general workflow (Figs. 1 and 2) and removed from the inverse PDMS moulds without leaving residues behind within the mould, as determined by PDMS mould inspection with fluorescence microscopy (Supplementary Fig. 1). Microscopic evaluation showed that, with the current methodology, intact and sharp microneedles could be made for the arrays containing different microneedle densities (Fig. 9a).

Next, piercing tests were performed on *ex vivo* human skin to assess the piercing ability of microneedles with varying densities (Fig. 9b). Within the range of densities tested, no significant differences were observed in penetration efficiency (Fig. 9c) or the geometry/dimensions of the skin pores (Fig. 9d). No significant difference was observed between the arrays, and therefore, no bed-of-nails effect was observed for the tested microneedle arrays. However, we observed a linear increase in the total skin pore surface area as the microneedle density increased (Fig. 9e).

The data and procedures obtained from this study can be instrumental in the future development of novel microneedle systems in several ways. Firstly, the established general workflow for producing polymeric microneedles utilising 2PP and PDMS moulds provides a robust foundation for fabricating strong and biocompatible microneedles. Secondly, the comprehensive toolbox of microneedle design parameters offers a valuable resource for optimising microneedle characteristics tailored to specific applications. Our methodology recognises that optimal microneedle designs must be adapted to the specific therapeutic context. Factors such as drug type, delivery depth, and treatment duration can significantly influence the optimal microneedle geometry and array configuration. Researchers can fine-tune microneedle arrays to maximise immune response and therapeutic outcomes by understanding how different design parameters affect skin penetration and micro-damage. For example, upon microneedle insertion, each microneedle causes a minor skin disruption, potentially resulting in a mechanically-induced adjuvant effect. The skin disruptions can trigger the release of danger-associated molecular patterns (DAMPs), which act as molecular adjuvants, amplifying the immune response to the administered antigen [52, 57]. This, in turn, can cause an increased potency of the vaccine at lower doses, which is especially important against weak antigens, such as tumour antigens. Altogether, the knowledge for this study will guide the development of advanced microneedle systems to achieve precise, skin-targeted delivery, enhancing the overall efficacy and safety of intradermal delivery of therapeutics.

## Conclusion

We successfully developed a general workflow for fabricating polymeric microneedles utilising 2PP and PDMS moulding. Our study demonstrates that microneedles made by this workflow can pierce human skin. More than 75 different designs were fabricated as master templates, varying one design parameter at a time, enabling systematic evaluation of individual parameters and their impact on skin piercing. This workflow facilitated the optimisation of microneedle design, where we observed that all microneedle base shapes perform equally well at sufficiently high insertion velocities, except obelisk-shaped microneedles, which showed significantly reduced piercing efficiencies. Furthermore, our findings indicate that the tip design becomes critical when insertion velocities fall below a certain threshold. In such cases, conical tips consistently demonstrated efficient penetration at reduced velocities, making them the preferred choice for applications where reduced impact speed is desirable or sophisticated microneedle applicators are unavailable. Our methodology acknowledges that optimal microneedle designs must be tailored to the specific therapeutic context, since factors such as drug type, delivery depth, and treatment duration can significantly influence the ideal microneedle geometry and array configuration. Additionally, from a translational perspective, it is also important to highlight that most materials used in our fabrication workflow are available in GMP-compliant or pharmaceutical-approved forms, facilitating potential clinical adoption. Altogether, the reliability of our fabrication methodology, coupled with the insights gained from *ex vivo* human skin experiments, enables follow-up research to optimise microneedle design, materials and formulations for specific intradermal immunotherapies (*e.g.*, cancer vaccination).

## Supplementary Information

Below is the link to the electronic supplementary material.

Supplementary file1 (DOCX 4671 KB)

## Data Availability

The datasets generated during and/or analysed during the current study are available from the corresponding authors on reasonable request.
