# Supplementary material for: A Comprehensive Design-to-skin Pipeline to Fabricate Polymeric Microneedles Using Ultrahigh-resolution 3D Printing
Source: Pharm Res. 2025 Nov 11;42(11):2125–43. doi: 10.1007/s11095-025-03936-x (PMC12698738; doi:10.1007/s11095-025-03936-x)
Supplement: Supplementary file 1 — Supplementary file1 (DOCX 4671 KB) [file 11095_2025_3936_MOESM1_ESM.docx]

***
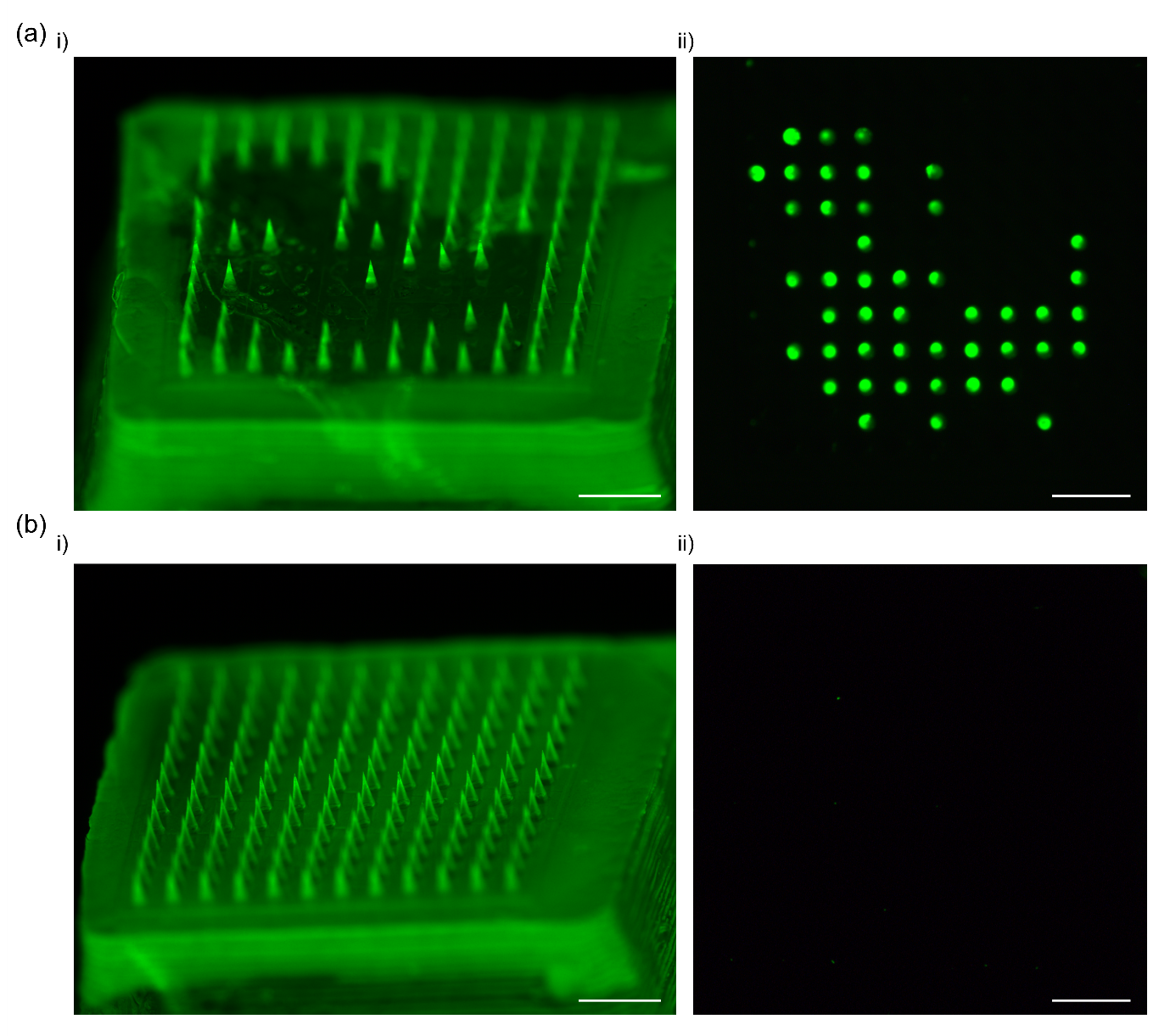
***

**Figure S1.** Polymeric microneedle arrays and inverse PDMS moulds after demoulding. Microneedle arrays (5 x 5 mm) consist of conical microneedles with a circular shape (length of 500 µm, tip diameter of 3 µm, base diameter of 200 µm). Microneedle arrays were imaged from a lateral view with an 80-degree angle. (a) (i) Incomplete microneedle array and (ii) the corresponding mould with residues. (b) Intact microneedle array and (ii) the corresponding mould without residues. Scale bars = 5 mm


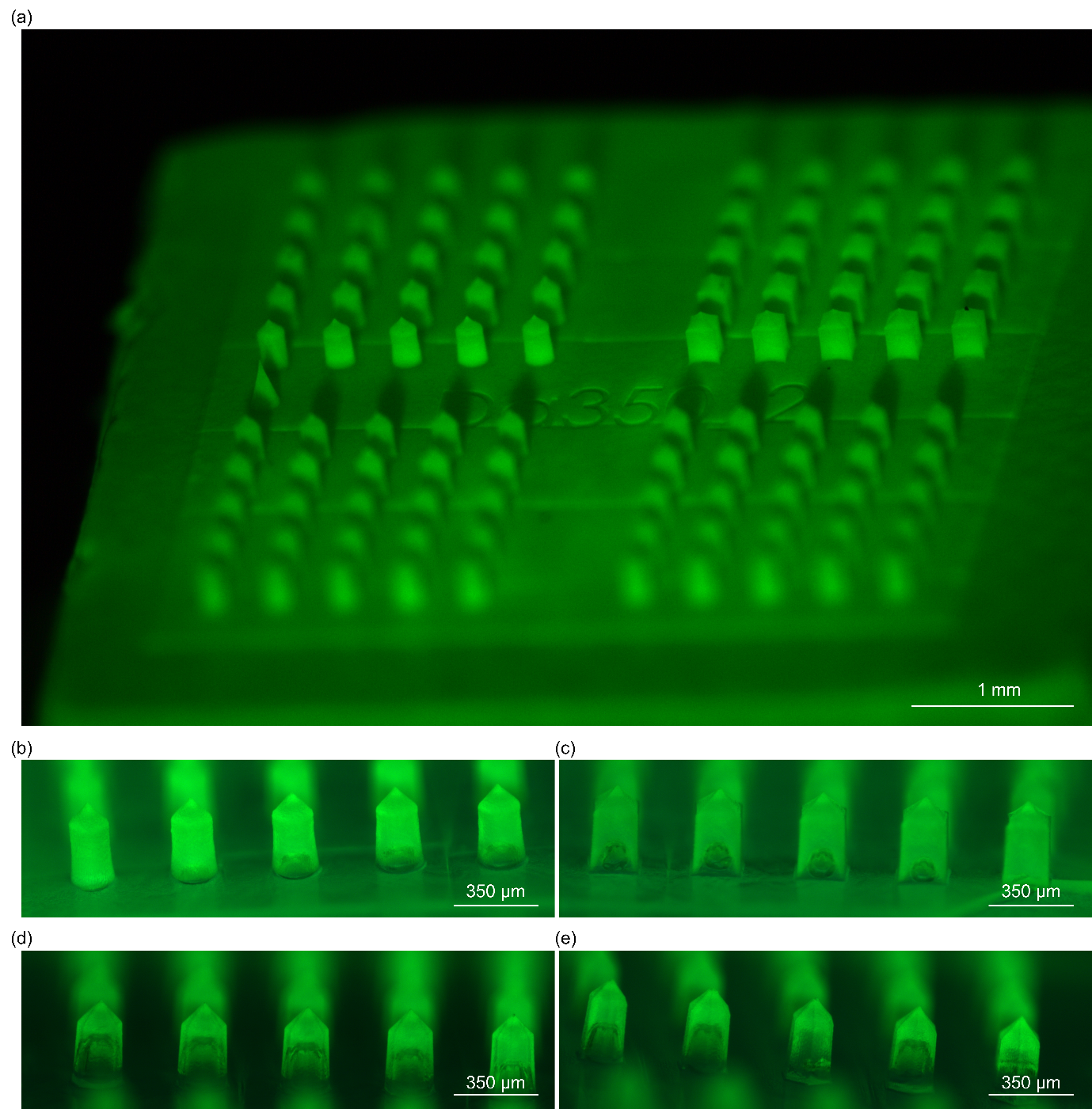


**Figure S2.** Stereomicroscopic fluorescence images of polymeric microneedle arrays. (a) Microneedle array (5 x 5 mm) include 25 microneedles of each shape: (b) circular, (c) tetragonal, (d) hexagonal and (e) octagonal, resulting in a total of 101 needles (including a reference microneedle). Microneedle arrays were imaged from a lateral view with an 80-degree angle. All microneedles had a tip diameter of 3 µm, base diameter of 200 µm, length of 350 µm and a pitch distance of 400 µm.

**Table SI.** Characteristic measurements of conical polymeric microneedle arrays for the microneedle designs with different tip diameters. CAD-based sizes: base diameter of 200 µm and length of 500 µm. Analysed with fluorescence stereomicroscopy, GFP filter set (excitation wavelength: 460-90 nm/emission wavelength: 510 nm). Data are shown as mean ± SD

| Geometry | Shape | Length (µm) | Diameter (µm) | Tip (µm) |
| --- | --- | --- | --- | --- |
| Cones  Tip 3 µm | Circular | 429 ± 4 | 182 ± 5 | 2,6 ± 0,3 |
|  | Tetragonal | 445 ± 6 | 181 ± 2 | 2,6 ± 0,4 |
|  | Hexagonal | 384 ± 4 | 178 ± 4 | 2,6 ± 0,3 |
|  | Octagonal | 434 ± 5 | 182 ± 1 | 2,9 ± 0,3 |
| Cones  Tip 10 µm | Circular | 404 ± 8 | 153 ± 1 | 9,8 ± 0,1 |
|  | Tetragonal | 428 ± 8 | 169 ± 4 | 10,1 ± 0,4 |
|  | Hexagonal | 354 ± 6 | 147 ± 3 | 10,1 ± 0,4 |
|  | Octagonal | 349 ± 5 | 147 ± 2 | 10,7 ± 0,4 |
| Cones  Tip 20 µm | Circular | 468 ± 4 | 166 ± 1 | 17,7 ± 0,8 |
|  | Tetragonal | 468 ± 6 | 187 ± 3 | 19,1 ± 1,1 |
|  | Hexagonal | 464 ± 6 | 184 ± 5 | 18,1 ± 1,3 |
|  | Octagonal | 437 ± 5 | 179 ± 5 | 18,5 ± 2,1 |
| Cones  Tip 30 µm | Circular | 472 ± 4 | 189 ± 2 | 30,3 ± 0,5 |
|  | Tetragonal | 468 ± 1 | 184 ± 3 | 30,6 ± 0,4 |
|  | Hexagonal | 470 ± 6 | 185 ± 4 | 28,6 ± 1,8 |
|  | Octagonal | 471 ± 5 | 168 ± 3 | 27,4 ± 0,7 |
| Cones  Tip 50 µm | Circular | 445 ± 2 | 149 ± 2 | 33,3 ± 2,2 |
|  | Tetragonal | 455 ± 3 | 163 ± 2 | 37,3 ± 2,0 |
|  | Hexagonal | 435 ± 8 | 145 ± 4 | 36,2 ± 2,7 |
|  | Octagonal | 433 ± 4 | 152 ± 6 | 36,8 ± 1,8 |

**Table SII.** Characteristic measurements of obelisk polymeric microneedle arrays for the needle designs with different tip diameters. CAD-based sizes: base diameter is 200 µm, length shaft is 400 µm and length tip of 100 µm. Analysed with fluorescence stereomicroscopy, GFP filter set (excitation wavelength: 460-90 nm/emission wavelength: 510 nm). Data are shown as mean ± SD

| Geometry | Shape | Length shaft (µm) | Length tip (µm) | Diameter (µm) | Tip  (µm) |
| --- | --- | --- | --- | --- | --- |
| Obelisks  Tip 3 µm  Length shaft 335 µm  Length tip 165 µm | Circular | 304 ± 5 | 131 ± 1 | 160 ± 6 | 2,8 ± 0,1 |
|  | Tetragonal | 306 ± 4 | 131 ± 3 | 166 ± 6 | 3,3 ± 0,4 |
|  | Hexagonal | 315 ± 4 | 130 ± 5 | 177 ± 5 | 3,1 ± 0,5 |
|  | Octagonal | 307 ± 3 | 126 ± 5 | 178 ± 6 | 3,2 ± 0,2 |
| Obelisks  Tip 10 µm  Length shaft 341 µm  Length tip 159 µm | Circular | 300 ± 3 | 153 ± 2 | 162 ± 4 | 8,6 ± 0,7 |
|  | Tetragonal | 292 ± 6 | 165 ± 3 | 170 ± 7 | 9,4 ± 0,6 |
|  | Hexagonal | 303 ± 9 | 154 ± 8 | 185 ± 4 | 9,2 ± 1,6 |
|  | Octagonal | 309 ± 2 | 160 ± 1 | 185 ± 3 | 8,9 ± 0,5 |
| Obelisks  Tip 20 µm  Length shaft 349 µm  Length tip 151 µm | Circular | 296 ± 7 | 157 ± 8 | 178 ± 5 | 17,6 ± 0,2 |
|  | Tetragonal | 297 ± 6 | 160 ± 3 | 188 ± 4 | 18,5 ± 0,9 |
|  | Hexagonal | 284 ± 3 | 151 ± 3 | 196 ± 1 | 18,7 ± 0,2 |
|  | Octagonal | 288 ± 6 | 138 ± 2 | 177 ± 5 | 18,5 ± 0,2 |
| Obelisks  Tip 30 µm  Length shaft 358 µm  Length tip 142 µm | Circular | 271 ± 6 | 145 ± 1 | 178 ± 7 | 24,6 ± 0,7 |
|  | Tetragonal | 275 ± 7 | 149 ± 8 | 176 ± 4 | 25,9 ± 0,8 |
|  | Hexagonal | 302 ± 2 | 130 ± 4 | 177 ± 2 | 23,6 ± 1,8 |
|  | Octagonal | 288 ± 5 | 132 ± 7 | 170 ± 6 | 23,3 ± 0,7 |
| Obelisks  Tip 50 µm  Length shaft 374 µm  Length tip 126 µm | Circular | 286 ± 9 | 135 ± 5 | 172 ± 5 | 33,7 ± 0,5 |
|  | Tetragonal | 307 ± 9 | 127 ± 5 | 178 ± 2 | 43,1 ± 2,1 |
|  | Hexagonal | 294 ± 9 | 128 ± 3 | 177 ± 4 | 34,4 ± 2,8 |
|  | Octagonal | 287 ± 6 | 120 ± 5 | 176 ± 1 | 34,9 ± 0,7 |

**Table SIII.** Characteristic measurements of conical polymeric microneedle arrays for the needle designs with different tip diameters. CAD-based sizes: tip diameter of 3 µm and length of 500 µm. Analysed with fluorescence stereomicroscopy, GFP filter set (excitation wavelength: 460-90 nm/emission wavelength: 510 nm). Data are shown as mean ± SD

| Geometry | Shape | Length (µm) | Diameter (µm) | Tip (µm) |
| --- | --- | --- | --- | --- |
| Cones  Base 150 µm | Circular | 218 ± 9 | 146 ± 3 | 5,4 ± 0,2 |
|  | Tetragonal | 232 ± 3 | 134 ± 4 | 3,5 ± 0,3 |
|  | Hexagonal | 219 ± 4 | 141 ± 2 | 4,2 ± 0,3 |
|  | Octagonal | 221 ± 2 | 141 ± 1 | 4,6 ± 0,3 |
| Cones  Base 200 µm | Circular | 233 ± 5 | 192 ± 3 | 4,7 ± 0,4 |
|  | Tetragonal | 244 ± 4 | 186 ± 5 | 3,5 ± 0,3 |
|  | Hexagonal | 238 ± 2 | 185 ± 2 | 3,4 ± 0,9 |
|  | Octagonal | 349 ± 5 | 147 ± 2 | 10,7 ± 0,4 |

| Geometry | Shape | Length shaft (µm) | Length tip (µm) | Diameter (µm) | Tip  (µm) |
| --- | --- | --- | --- | --- | --- |
| Obelisks  Total length 250 µm  Length shaft 150 µm  Length tip 100 µm | Circular | 124 ± 4 | 117 ± 3 | 167 ± 3 | 2,9 ± 0,2 |
|  | Tetragonal | 122 ± 5 | 118 ± 2 | 172 ± 1 | 3,0 ± 0,1 |
|  | Hexagonal | 125 ± 4 | 106 ± 3 | 176 ± 1 | 3,1 ± 0,4 |
|  | Octagonal | 126 ± 3 | 111 ± 3 | 174 ± 4 | 2,8 ± 0,1 |
| Obelisks  Total length 350 µm  Length shaft 250 µm  Length tip 100 µm | Circular | 195 ± 6 | 112 ± 2 | 167 ± 6 | 2,7 ± 0,3 |
|  | Tetragonal | 206 ± 8 | 109 ± 5 | 176 ± 2 | 2,9 ± 0,2 |
|  | Hexagonal | 212 ± 2 | 98 ± 4 | 180 ± 5 | 2,9 ± 0,3 |
|  | Octagonal | 210 ± 4 | 101 ± 4 | 164 ± 8 | 3,1 ± 0,3 |
| Obelisks  Total length 500 µm  Length shaft 400 µm  Length tip 100 µm | Circular | 304 ± 5 | 131 ± 1 | 160 ± 6 | 2,8 ± 0,1 |
|  | Tetragonal | 306 ± 4 | 131 ± 3 | 166 ± 6 | 3,3 ± 0,4 |
|  | Hexagonal | 315 ± 4 | 130 ± 5 | 177 ± 5 | 3,1 ± 0,5 |
|  | Octagonal | 307 ± 3 | 126 ± 5 | 178 ± 6 | 3,2 ± 0,2 |
| Obelisks  Total length 650 µm  Length shaft 550 µm  Length tip 100 µm | Circular | 444 ± 5 | 115 ± 4 | 180 ± 8 | 2,8 ± 0,1 |
|  | Tetragonal | 435 ± 7 | 112 ± 1 | 168 ± 4 | 2,8 ± 0,1 |
|  | Hexagonal | 432 ± 7 | 99 ± 3 | 175 ± 3 | 3,0 ± 0,3 |
|  | Octagonal | 432 ± 10 | 101 ± 5 | 170 ± 7 | 2,8 ± 0,5 |

**Table SIV.** Characteristic measurements of obelisk polymeric microneedle arrays for the needle designs with different tip diameters. CAD-based sizes: tip diameter of 3 µm and base diameter of 200 µm. Analysed with fluorescence stereomicroscopy, GFP filter set (excitation wavelength: 460-90 nm/emission wavelength: 510 nm). Data are shown as mean ± SD


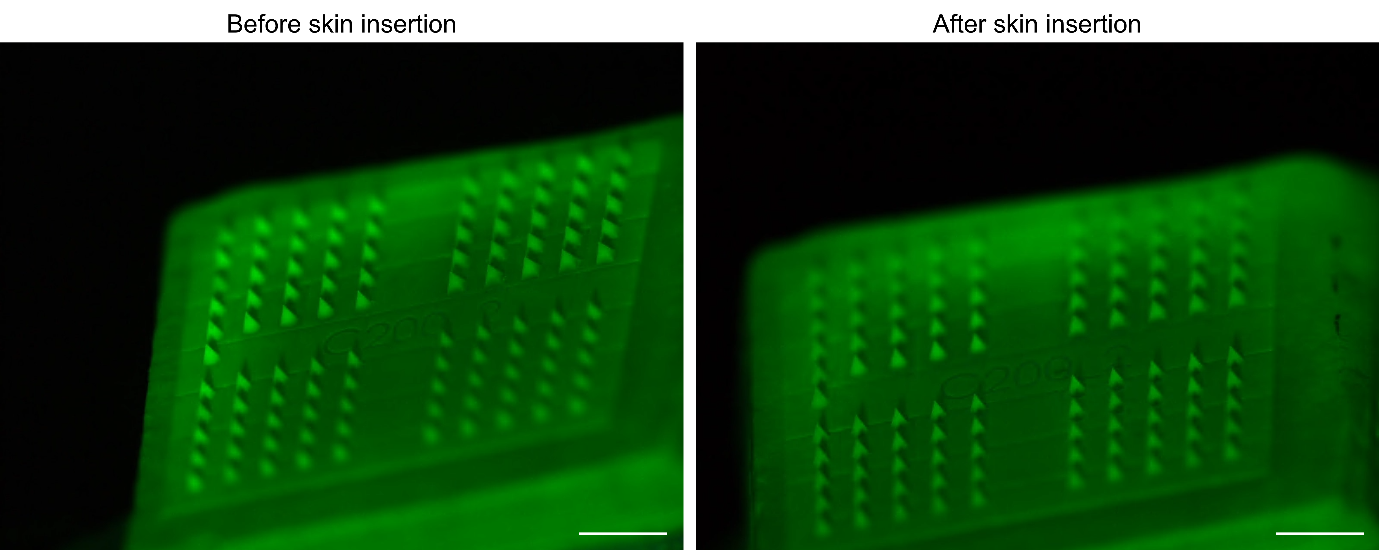


**Figure S3.** Stereomicroscopic fluorescence images of polymeric microneedle arrays before and after skin insertion. All microneedles had a tip diameter of 3 µm, base diameter of 200 µm, length of 500 µm and a pitch distance of 400 µm. Scale bares = 1 mm
